# Defining the role of multiparametric MRI in predicting prostate cancer extracapsular extension

**DOI:** 10.1007/s00345-023-04720-5

**Published:** 2024-01-13

**Authors:** Francesco Sanguedolce, Alessandro Tedde, Luisa Granados, Jonathan Hernández, Jorge Robalino, Edgar Suquilanda, Matteo Tedde, Joan Palou, Alberto Breda

**Affiliations:** 1 Department of Urology, Fundació Puigvert, Autonoma University of Barcelona, Barcelona, Spain; 2 Department of Urology, Università degli Studi di Sassari, Sassari, Italy; 3 Department of Radiology, Fundació Puigvert, Autonoma University of Barcelona, Barcelona, Spain; 4 Department of Medicine, Surgery and Pharmacy, Universitá degli Studi di Sassari, Sassari, Italy; 5 Department of Urology, Fundació Puigvert, Barcelona, Spain; 6 Institut Reserca Sant Pau, Institut Reserca Sant Pau, Barcelona, Spain

**Keywords:** Prostate cancer, Multiparametric magnetic resonance imaging, Radical prostatectomy, Extracapsular extension, Predictive models, Nomogram

## Abstract

**Objectives:**

To identify the predictive factors of prostate cancer extracapsular extension (ECE) in an institutional cohort of patients who underwent multiparametric MRI of the prostate prior to radical prostatectomy (RP).

**Patients and methods:**

Overall, 126 patients met the selection criteria, and their medical records were retrospectively collected and analysed; 2 experienced radiologists reviewed the imaging studies. Logistic regression analysis was conducted to identify the variables associated to ECE at whole-mount histology of RP specimens; according to the statistically significant variables associated, a predictive model was developed and calibrated with the Hosmer–Lomeshow test.

**Results:**

The predictive ability to detect ECE with the generated model was 81.4% by including the length of capsular involvement (LCI) and intraprostatic perineural invasion (IPNI). The predictive accuracy of the model at the ROC curve analysis showed an area under the curve (AUC) of 0.83 [95% CI (0.76–0.90)], *p* < 0.001. Concordance between radiologists was substantial in all parameters examined (*p* < 0.001). Limitations include the retrospective design, limited number of cases, and MRI images reassessment according to PI-RADS v2.0.

**Conclusion:**

The LCI is the most robust MRI factor associated to ECE; in our series, we found a strong predictive accuracy when combined in a model with the IPNI presence. This outcome may prompt a change in the definition of PI-RADS score 5.

**Supplementary Information:**

The online version contains supplementary material available at 10.1007/s00345-023-04720-5.

## Introduction

Multiparametric MRI (mpMRI) of the prostate is nowadays considered the key tool for the diagnosis of clinically significant prostate cancer (csPCa) [1].

The quality of MRI has progressively increased in the last years and standardised multiparametric sequences acquisition and reporting have been established, with the development of the Prostate Imaging – Reporting and Data System (PI-RADS) scoring system, first published in 2012 by the European Society of Urogenital Radiology (ESUR) [2].

The updated PI-RADS versions 2 and 2.1 are nowadays broadly used in clinical practice, overcoming some ambiguities and limitations related to the overall scoring system of the former version [3, 4].

Nevertheless, PI-RADS has not being designed for staging purposes; on this regard, the ESUR developed another score system based on capsular alterations detectable at MRI and related to the likelihood of an extracapsular extension (ECE) of the lesion [5].

During treatment planning, the identification of ECE as a strategy for local staging is crucial in order to achieve an adequate balance between cancer control and preservation of potency and continence, ultimately obtaining the best surgical, oncological and functional results.

The main objective of this study was to identify clinical, pathological and radiological parameters associated to ECE in a single institution cohort of patients undergoing mpMRI prior to radical prostatectomy (RP), as by whole-mount prostate sections for definitive histological assessment. Furthermore, we evaluated the usefulness and the inter-observer variability of the overall LIKERT (subjective operator assessment for the likelihood of a csPCa, from 1 to 5), ECE-LIKERT (subjective operator assessment for the likelihood of ECE, from 1 to 5), PI-RADS v2 and ESUR-ECE, in order to evaluate their performance in predicting the ECE risk in the same cohort of patients.

## Patients and methods

### Study design and population

This is a retrospective analysis of patients who had undergone mpMRI before RP at Fundació Puigvert – Barcelona (ES), between April 2013 (date of the implementation of mpMRI driven pathway) and December 2017. The mpMRI was requested at urology consultant discretion, either before or after the diagnostic biopsy, as no specific recommendations for requesting an mpMRI were still put in place during the period in observation nor in the international guidelines neither in our internal protocol.

Overall, 126 patients met the selection criteria (mpMRI undertaken within 6 months before surgery, either before or after the diagnostic biopsy; availability of full set of data in observation) and their medical records were collected and reviewed. The patients’ data were managed according to our institutional review board protocol, in full compliance with both the principles of the latest version of the Declaration of Helsinki and of the Spanish adaptation of the General Data Protection Regulation (organic law 3/2018, December the 5th 2018).

### mpMRI protocol

A 3-Tesla mpMRI examination with a pelvic phased-array surface coil was performed for all patients. The mpMRI protocol-included T2-weighted (T2W) sequences in three planes, Diffusion Weighted Imaging (DWI) sequences with high *b*-values (> 1200 s/mm^2^) and apparent diffusion coefficient (ADC) map, and Dynamic Contrast-Enhanced (DCE) sequences with a bolus of gadolinium contrast medium injection. Imaging acquisition protocol was rigorously compliant with the PIRADS v1 guidelines, in force during the period in observation [2]. It is Important to note that these guidelines also included recommendations for waiting a period of 4–6 weeks between a biopsy and the eventual MRI to minimise the effect of eventual haemorrhage, and in case of substantial persisting artifacts to repeat the MRI within further 4 weeks or so. These recommendations were duly followed, and interestingly remained substantially unvaried along the updated v2 and v2.1 versions [3, 4]. All images were retrospectively and independently reassessed by two expert radiologists (L.G. and J.H.) with at least 4 years of experience in prostate mpMRI, both blinded to clinical and histological data; PI-RADS v2 was used to score the MRI explorations, as the updated v2.1 was published posteriorly to the radiological revision of the imaging tests. The maximal index lesion size (ILS), length of capsular involvement (LCI) by tumour, number of lesions and location were recorded whenever visible from the dominant sequence involved. The radiologists also used an overall-LIKERT and ECE-LIKERT scores, as by subjective impression of the likelihood of significant malignancy and extracapsular extension (1 = very unlikely; 5 = very likely), according to the recommendation of the PREDICT (Prostate Diagnostic Imaging Consensus Meeting) panel [6]. Furthermore, the likelihood of ECE was evaluated using the ESUR MRI scoring guidelines of extra-prostatic disease [5].

### Reference standard

Whole-mount histological sections from the RP specimens were used as the reference standard. The specimens were fixed in 10% buffered formalin, and sectioned into horizontal sections of 3–4 mm. All tissues were paraffin-embedded, and 3–4 microns sections were obtained and stained with hematoxylin–eosin; then, the sections of the tissue were assessed by a single expert uropathologist (F.A.), blinded to mpMRI data. The uropathologist recorded cancer location, size, volume, and Gleason grade group according to the International Society of Urological Pathology (ISUP) consensus conference of 2014 [7].

### Outcomes and statistical analysis

The primary outcome consisted in identifying the parameters associated to the ECE at the RP specimen. Descriptive data were expressed as median and interquartile range (IQR, 25–75 quartile). Analysis between groups was performed using Student’s *t* test (Mann–Whitney *U* test in variables without normal distribution) for continuous variables, and Chi-square (Fisher’s exact test with observed frequencies < 5) for categorical variables.

Quantitative (continuous) variables were transformed to binary (categorical) variables before inclusion in logistic models using the best predictive cut-off point obtained with Receiver Operating Characteristics (ROC) curve analysis.

Univariate and multivariate logistic regression models were performed including ECE as a dependent variable. Preoperative clinical, pathological and mpMRI variables with *p* value < 0.2 at the univariate analysis were included as independent variables using the backward stepwise logistic regression analysis. Predictors from the final model were used to calculate the likelihood of ECE according to the following equation: Exp(*β*)/[1 + Exp(*β*)], where *β* = [− 3.00 + X*(predictor *A*) + Y*(predictor *B*)] for two predictors. The quality of the final model was assessed using the Hosmer–Lemeshow goodness-of-fit-test.

Inter-observer agreement between the two radiologists regarding the MRI features/scores was assessed using the intraclass correlation coefficient (ICC) with the 95% confidence interval, applying a two-way ICC with a random rater assumption. The agreement (match) between a PCa lesion detected at mpMRI and an equivalent lesion at whole-mount histological sections from the RP specimens was assessed using Cohen Kappa coefficient, whose results were categorised in standardised ranges  < 0.4 poor agreement; 0.4–0.6 moderate agreement; 0.61–0.8 substantial agreement; 0.81–1 as excellent. A *p*-value < 0.05 was considered statistically significant for all cases. Statistical analysis was performed using R studio (V2.5) package.

## Results

The clinical, bioptic and MRI features are summarised in Table 1. The median age at prostate biopsy, PSA and PSA-density were 66.6 years, 7.2 ng/ml and 0.2 ng/ml^2^, respectively.

**Table 1:** Patients’ clinical data

Variables	Median (IQR)	*N* (%)
Age at biopsy (yr)	66.6 (61.5–68.9)	
Clinical stage		
T1		85 (67.5)
T2 + T3		41 (32.5)
Baseline PSA at biopsy (ng/ml)	7.2 (5.4–10.4)	
Max length core (mm)	5 (3–8)	
Intraprostatic perineural invasion		
Yes		26 (20.6)
No		100 (79.4)
Prostatic volume Observer #1 (cc)	45.5 (32–65.2)	
Prostatic volume Observer #2 (cc)	50 (35–74)	
PSA-density at biopsy (ng/cc)	0.17 (0.11–0.26)	
Number of targeted biopsies (n. 49, 38.89%)	3 (3–4)	
Number of positive targeted biopsy (n. 49, 38.89%)	2 (0–3)	
ISUP group at biopsy		
1		41 (32.5)
2		47 (37.3)
3		9 (7.1)
4		18 (14.3)
5		11 (8.8)
Type of MRI		
T2w + DWI + DCE		123 (97.6)
T2w + DWI		3 (2.4)
Index Lesion size (mm), Observer #1 Median (IQR)	12 (8–19)	
Index Lesion size (mm), Observer #2 Median (IQR)	12 (8–18)	
Length of capsular involvement (mm) Median (IQR)	9 (3.7–15.2)	

*yr* years, *ng* nanograms, *ml* millilitres, *mm* millimetres, *cc* cubic centimetres

Overall, 41 patients (32.5%) had T2 or T3 clinical stage. Intraprostatic perineural invasion (IPNI) and ISUP group grade > 3 were observed in 26 (20.6%) and 29 (23%) patients, respectively.

The median index lesion size (ILS) and length of capsular involvement (LCI) were 12 and 9 mm (mm), respectively. MRI readings based on PI-RADS v2, ESUR and LIKERT scores are available in the Supplementary material.

Pathology data and ECE analysis are summarised in Table 2 and Supplementary material, respectively. The median ILS of RP specimen was 16 mm. Accordingly, MRI underestimated the ILS by a 25% in comparison to the true specimen size.

**Table 2:** Patients’ pathology data (*N* = 126 patients)

Variables	Median (IQR)	*N* (%)
Index Lesion size (mm)	16 (10–21)	
Histology		
pT0		2 (1.6)
pT2a		25 (19.9)
pT2b		8 (6.3)
pT2c		56 (44.4)
pT3a		27 (21.4)
pT3b		8 (6.4)
ISUP grade group		
0		2 (1.6)
1		15 (11.9)
2		51 (40.5)
3		18 (14.4)
4		23 (18.2)
5		17 (13.4)

*mm* millimetres

Overall, ECE was found in 35 (27.8%) patients; definitive ISUP grade > 3 was observed in 40 (31.7%) patients.

The following variables were significantly associated to ECE: length of biopsy core (LBC; median: 7 vs 4 mm, *p* < 0.001), intraprostatic perineural invasion (48.6% vs 9.9%, *p* < 0.001), ILS (median: 20 vs 10 mm, *p* < 0.001), LCI (median: 17 vs 7 mm, *p* < 0.001), PSA density (0.24 vs 0.14, *p* = 0.001), and biopsy ISUP grade > 3 (40% vs 16.4%, *p* = 0.003).

At ROC Curve analysis, the best cut-off points for LBC, LCI and ILS were identified as 5.5, 9.5 and 11 mm, respectively.

**Fig. 1:**
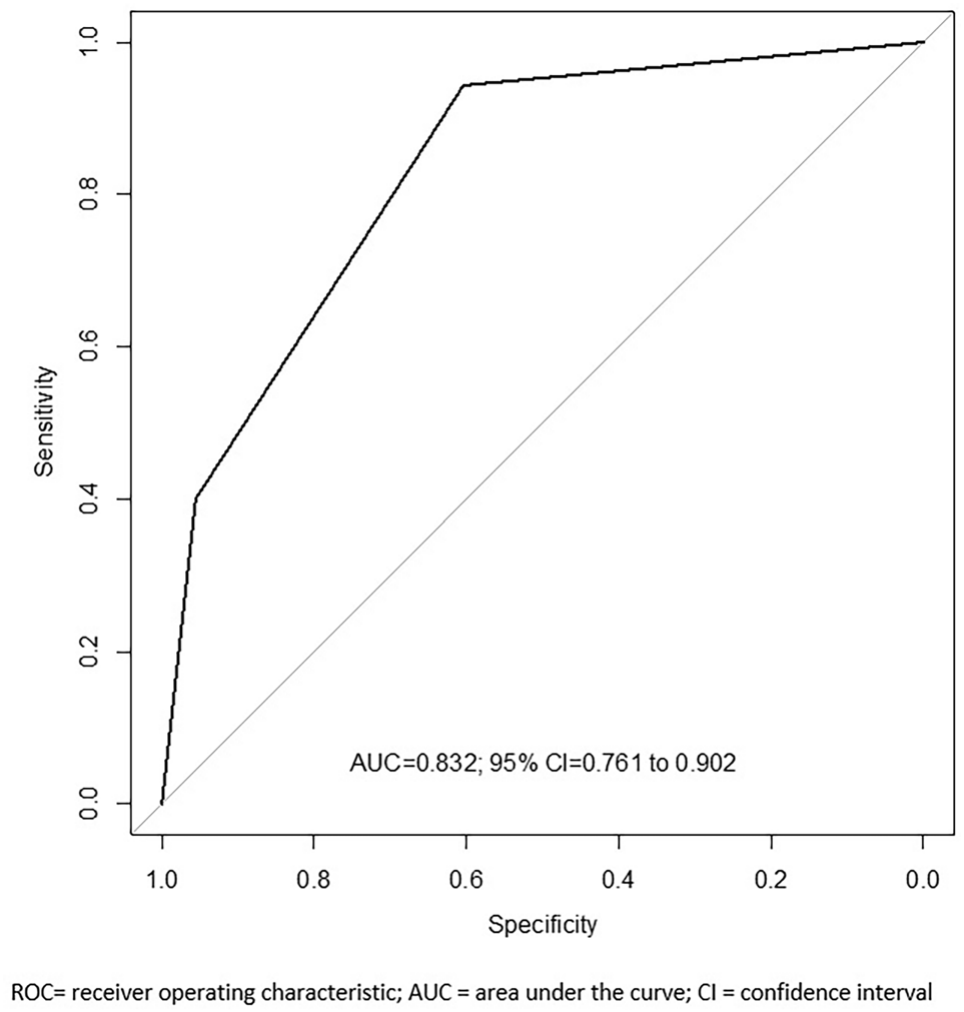
ROC curve analysis for predictive model

Overall, both LCI and IPNI showed statistical significance (*p* < 0.001) at multivariate logistic regression model (Table 3). The probability to detect ECE with the generated model was 81.4% by including the two variables (Supplementary material). The model was calibrated with an overall p-value of 0.985 by using the Hosmer–Lemeshow test (*R*^2^ = 66%), and the predictive accuracy was evaluated through ROC curve analysis, with an area under the curve (AUC) of 0.83 [95% CI (0.76–0.90)], *p* < 0.001 (Fig. 1).

**Table 3:** Logistic Regression for ECE

	Univariate			Multivariate			Multivariate*		
	OR	95% CI	*p* value	OR	95% CI	*p* value	OR	95% CI	*p* value
Clinical stage									
T1	Ref	–	***0.002***	Ref	–	*0.971*			
T2 + T3	3.72	1.65–8.58		1.02	0.31–3.22				
PSA density (ng/ml^2^)									
≤ 0.15	Ref	–	***0.003***	Ref	–	*0.406*			
> 0.15	4.16	1.71–11.29		1.75	0.48–6.97				
Max length core (mm)									
≤ 5.5	Ref	–	** < *0.001***	Ref	–	*0.096*			
> 5.5	5.93	2.57–14.55		2.85	0.85–10.36				
IPNI									
No	Ref	-	** < *0.001***	Ref	–	***0.001***	Ref	–	** < *0.001***
Yes	8.61	3.31–22.37		10.24	2.57–40.85		8.17	2.78–26.32	
ISUP grade at biopsy									
≤ 3	Ref	–	** < *0.001***	Ref	–	*0.078*			
> 3	4.39	1.93–10.66		2.81	0.91–9.37				
LCI (mm)									
≤ 9.5	Ref	–	** < *0.001***	Ref	–	***0.002***	Ref	–	** < *0.001***
> 9.5	11.61	4.41–36.77		14.34	2.87–95.10		11.09	3.91–38.18	
ILS at MRI (mm)									
≤ 11	Ref	–	** < *0.001***	Ref	–	*0.673*			
> 11	8.86	3.38–27.96		0.69	0.12–3.81				

**Multivariate**: Analysis conducted considering all parameters; Multivariate*: Analysis conducted considering all parameters with stepwise method for statistical significance (*p* < 0.05)

*ECE* extracapsular extension, *OR* odds-ratio, *IPNI* intraprostatic perineural invasion, *LCI* length of capsular involvement, *ILS* index lesion size

Bold Italic are reported the statistically significant *p*-values

Univariate and multivariate logistic regression analyses were also conducted to identify predictors in the subgroup of patients with seminal vesicle invasion (SVI), but they were not included in the final model because of the low number of events (*n* = 8). Data analysis and comparison of ROC curve between final model and imaging scores models (PI-RADS v2, LIKERT, ESUR) are available in the Supplementary material.

Concordance between the two radiologists was evaluated for ILS, PI-RADS v2, LIKERT and ESUR score, with an overall ICC > 0.6 (*p* < 0.001) in all parameters examined (Supplementary material). Correlation for ILS was 0.66 and 0.62 for L.G. and J.H., respectively (*p* < 0.001).

## Discussion

The prognosis of PCa is highly related to tumour stage, and ECE is the most common adverse feature found in histopathology after RP [8]. Inadequate selection of patients with ECE to nerve-sparing RP increases the risk of positive surgical margins with subsequent need for either adjuvant or salvage radiotherapy. In order to improve preoperative risk assessment, numerous nomograms have been developed in the last years, mostly based on the association of multiple clinical risk factors (PSA, biopsy ISUP grade, clinical stage, etc.) [9–12].

The Partin tables were among the first tools developed for the prediction of pathological stage, and have been widely used for several years for surgical planning [10]. Other predictive tools were subsequently developed using different variables (e.g. percentage of positive biopsy cores), but none of them reached a comparable popularity as for the Partin tables [9, 12]. Nevertheless, they could not distinguish unilateral from bilateral ECE [13].

With the advent of mpMRI, further nomograms have been developed in an attempt to improve the accuracy to predict the ECE. Feng et al. first reported that mpMRI could improve the performance of Partin tables and MSKCC nomogram regarding ECE prediction [14].

Giganti et al. developed a nomogram exploiting clinical and MRI parameters with strong accuracy for ECE prediction [15], subsequently confirmed by an external validation conducted by Alves et al. [16]. One of the most important features of their model was the excellent concordance between MRI-tumour volume and the ADC map of tumour lesion.

More recently, Gandaglia et al. developed a model to predict ECE, SVI and stage upgrading in patients diagnosed with MRI-targeted and concomitant systematic biopsies [17], achieving an AUC of 73% (ECE), 81% (SVI) and 73% (upgrading) at internal validation.

Nevertheless, a meta-analysis of de Rooij et al. reported high specificity but low sensitivity for mpMRI accuracy in local staging; overall, staging based on MRI alone lacks sensitivity in detecting ECE, especially in case of focal, minimal or microscopic extension because of limitation in spatial resolution [18, 19]. Moreover, the degree of underestimation increases with smaller radiologic tumour size and lower PI-RADS scores [20].

The MRI and histology biopsy features have been variably reported in literature in the recent past as predictors of ECE [21].

Baco et al. found that MRI-tumour LCI well correlated with ECE, with an AUC that outperformed the Partin tables; they also found higher accuracy for microscopic-ECE detection with a 20 mm threshold [22].

Similarly, Kongnyuy et al. identified MRI-LCI as a promising predictor of ECE, positive pathological lymph nodes and biochemical recurrence. The 12.5 mm cut-off showed the highest sensitivity (77%) and specificity (59%) in predicting ECE. The AUC was comparable to that of the Partin tables, outperforming them with LCI and PSA combination [23].

Moreover, in a recent meta-analysis of Li et al., the LCI showed high diagnostic performance in predicting ECE, with a pooled sensitivity and specificity of 0.79 and 0.77, respectively. When subgroup analysis was performed comparing different threshold values, lower LCI cut-off values yielded slightly better sensitivity and comparable specificity, without substantial differences between sub-groups [24].

In addition to LCI, IPNI is another acknowledged parameter often associated with ECE, possibly because in the 85% of cases the ECE goes through neurovascular bundles by dissection of the intraprostatic perineural spaces for tissue planes of least resistance [25]

Perineural invasion is defined as the tumour invasion into the perineural sheath, and during the years, it has been associated with tumour progression and prognosis of several malignancies, including prostate cancer [26]. In a recent study on upper urinary tract urothelial carcinomas, Lin et al. found that PNI-positive patients had unfavourable pathological features, including high pathological stage, high tumour grade and lymphovascular invasion, leading to worse progression-free survival (PFS) [26].

Algaba et al. found that IPNI is correlated to cancer volume and higher percentage of extraprostatic cancer [27]. Same finding was reported more recently by Leyh-Bannurah et al., being IPNI the only histological parameter found significantly associated to ECE in their nomogram [28] Nevertheless, IPNI has not yet been reported among the most powerful histological features even in the latest version of the EAU guidelines, so that our finding might prompt its inclusion among the reporting recommendations for the prostatic biopsy.

In our study, we identified both the LCI and the IPNI as predictors of ECE: the LCI cut-off that best correlated to ECE was 9.5 mm, which was a similar finding reported also by Li et al. [24].

Interestingly, in our cohort, the ILS at MRI did not show the same degree of association to the ECE as by the LCI; furthermore, MRI underestimated pathological tumour size by 25%.

Overall, these data may have several implications: (1) Prostate mpMRI alone, including singular features and scoring systems, do not adequately predict ECE, except LCI—especially in combination to a histology biopsy feature, as IPNI in our series. (2) A change of PI-RADS score 5 definition should be prompted in the future PI-RADS updated version: score 5 is attributed to ILS ≥ 15 mm, but this threshold was chosen by the PI-RADS steering committee on the basis of old studies of the’90, when csPCa and ECE were found to be correlated to a tumour volume ≥ 0.5 cm^3^ (15 mm in major axis) at whole-mount RP specimen [29, 30]. If differences of PI-RADS scores 4 to 5 are to be based on the risk of ECE, LCI should be the preferred MRI feature and with a lower cut-off, as the 15 mm cut-off at MRI might underestimate for a quarter the actual tumour size at specimen. (3) We found excellent or substantial inter-readers agreement for relevant MRI variables, thus strengthening the importance of high-quality imaging acquisition and readers’ skills for their adequate assessment. This latter matter has been popularised with the introduction of a dedicate score (PIQUAL) about the quality of the MRI sequences and the ability to make decision on the basis of it [31].

Main limitations of the study includes (1) the retrospective design of our analysis, even though significant efforts have been done in reviewing MRI images by two radiologists blinded to final histology; (2) the number of cases is limited, especially because MRI implementation in the clinical practice has substantially increased in more recent years; (3) the MRI images were reassessed according to the PI-RADS v2, as the version 2.1 became available on a later stage to that phase of our study. Nevertheless, it is very unlikely that the minor changes in score reporting of the latest version would have had an impact on the outcome of our study, as shown in a recent publication comparing the v2.0 vs v2.1 diagnostic performance with no difference in concordance rates between targeted biopsy and radical prostatectomy (doi: 10.2214/AJR.23.29964).

## Conclusions

The mpMRI confirms limitations in predicting the ECE at the RP specimen, even when involving tailored scoring systems. On the other side, MRI-LCI is the most robust factor associated to ECE; in our series, we found a strong predictive accuracy when combined with the IPNI presence. This outcome may prompt a change in the definition of PI-RADS score 5, by reducing IL size cut-off to 10–12mm, or by replacing IL size reporting with LCI (cut-off 9–10mm).

## Supplementary Information

Below is the link to the electronic supplementary material.

Supplementary file1 (DOCX 129 KB)
