# Supplementary material for: Defining the role of multiparametric MRI in predicting prostate cancer extracapsular extension
Source: World J Urol. 2024 Jan 13;42(1):37. doi: 10.1007/s00345-023-04720-5 (PMC10787875; doi:10.1007/s00345-023-04720-5)
Supplement: Supplementary file 1 — Supplementary file1 (DOCX 129 KB) [file 345_2023_4720_MOESM1_ESM.docx]

This supplementary material has been provided by the authors to give readers additional

information about their work.

**List of materials**

**eTable 1 – MRI data**

**eTable 2 – Univariate analysis for ECE**

**eTable 3 – Calibration of the predictive model for ECE**

**eTable 4 – Radiologists’ concordance**

**eFigure 1 – ROC Curve analysis, comparison between final model and PI-RADS/LIKERT/ESUR models**

**Legend**:

**ECE**: extracapsular extension

**MRI**: magnetic resonance imaging

**SVI**: seminal vesicle invasion

**ROC**: receiver operating characteristics

**eTable 1 – MRI data**

| **Variables** | | **N (%)** |
| --- | --- | --- |
| **PI-RADS v2, L.G.**  **(n. 125, 99.21%)** | **1** | 3 (2.4) |
|  | **2** | 17 (13.6) |
|  | **3** | 12 (9.6) |
|  | **4** | 44 (35.2) |
|  | **5** | 49 (39.2) |
| **PI-RADS v2, J.H.**  **(n. 124, 98.41%)** | **1** | 0 (0) |
|  | **2** | 14 (11.3) |
|  | **3** | 12 (9.7) |
|  | **4** | 53 (42.7) |
|  | **5** | 45 (36.3) |
| **ESUR score, L.G.**  **(n. 123, 97.62%)** | **0** | 0 (0) |
|  | **1** | 85 (69.1) |
|  | **2** | 3 (2.4) |
|  | **3** | 11 (8.9) |
|  | **4** | 21 (17.1) |
|  | **5** | 3 (2.4) |
| **ESUR score, J.H.**  **(n. 125, 99.21%), n (%)** | **0** | 0 (0.0) |
|  | **1** | 80 (64.0) |
|  | **2** | 2 (1.6) |
|  | **3** | 15 (12.0) |
|  | **4** | 21 (16.8) |
|  | **5** | 7 (5.6) |
| **LIKERT ECE, L.G.**  **(n.125, 99.21%), n (%)** | **1** | 62 (49.6) |
|  | **2** | 20 (16.0) |
|  | **3** | 15 (12.0) |
|  | **4** | 24 (19.2) |
|  | **5** | 4 (3.2) |
| **LIKERT ECE, J.H.**  **(n.124, 98.41%), n (%)** | **1** | 70 (56.4) |
|  | **2** | 7 (5.6) |
|  | **3** | 19 (15.3) |
|  | **4** | 21 (16.9) |
|  | **5** | 7 (5.6) |
| **Overall LIKERT, L.G.**  **(n. 125, 99.21%), n (%)** | **1** | 0 (0.0) |
|  | **2** | 19 (15.2) |
|  | **3** | 14 (11.2) |
|  | **4** | 38 (30.4) |
|  | **5** | 54 (43.2) |
| **Overall LIKERT, J.H.**  **(n. 124, 98.41%), n (%)** | **1** | 0 (0.0) |
|  | **2** | 14 (11.3) |
|  | **3** | 19 (15.3) |
|  | **4** | 35 (28.2) |
|  | **5** | 56 (45.2) |

**eTable 2 – Univariate analysis for ECE**

| **Variables** | | **ECE Presence**  **(n. 35)** | **ECE Absence**  **(n. 91)** | **p-value** |
| --- | --- | --- | --- | --- |
| **Age at biopsy (yr)**  **Median (IQR)** | | 66.6 (62.2 – 68.9) | 66.6 (61 – 69) | 0.903 |
| **Clinical Stage**  **N (%)** | **T1** | 16 (45.7) | 69 (75.8) | **0.003** |
|  | **T2 + T3** | 19 (54.3) | 22 (24.2) |  |
| **Baseline PSA at biopsy (ng/ml)**  **Median (IQR)** | | 9.2 (5.9 – 12) | 7 (5.2 – 10) | 0.106 |
| **Max length core (mm)**  **Median (IQR)** | | 7 (5 – 11) | 4 (2 – 6) | **<0.001** |
| **Intraprostatic perineural invasion** | **Yes** | 17 (48.6) | 9 (9.9) | **<0.001** |
|  | **No** | 18 (51.4) | 82 (90.1) |  |
| **Index Lesion size (mm), Observer #1**  **Median (IQR)** | | 20 (13.7 – 23) | 10 (6 – 17) | **<0.001** |
| **Index Lesion size (mm), Observer #2**  **Median (IQR)** | | 19 (12.7 – 23.2) | 10 (7.5 – 15) | **<0.001** |
| **Length of capsular involvement (mm),**  **Median (IQR)** | | 17 (10 – 22) | 7 (0 – 12) | **<0.001** |
| **Prostatic volume, (cc), Observer #1**  **Median (IQR)** | | 35 (24 – 53.5) | 50 (34.5 – 70.5) | **0.004** |
| **Prostatic volume (cc), Observer #2**  **Median (IQR)** | | 39 (29.5 – 57.5) | 56 (41.5 – 81) | **0.009** |
| **PSA-density at biopsy (ng/ml^2^)**  **Median (IQR)** | | 0.2 (0.2 – 0.3) | 0.1 (0.1 – 0.2) | **0.001** |
| **Number of targeted biopsies**  **(n. 49, 38.89%) Median (IQR)** | | 3 (3 – 4) | 3 (3 – 4) | 0.691 |
| **Number of positive targeted biopsy**  **(n. 49, 38.89%) Median (IQR)** | | 3 (1.2 – 3.7) | 1 (0 – 3) | **0.034** |
| **ISUP group grade at biopsy** | **1** | 2 (5.71) | 39 (42.86) | **0.003** |
|  | **2** | 15 (42.86) | 32 (35.16) |  |
|  | **3** | 4 (11.43) | 5 (5.49) |  |
|  | **4** | 6 (17.14) | 12 (13.19) |  |
|  | **5** | 8 (22.86) | 3 (3.30) |  |

yr= years; ng= nanograms; ml=millilitres; mm = millimetres; cc= cubic centimetres; ECE= extracapsular extension

**eTable 3 – Calibration of the predictive model for ECE**

| **Variables** | **ECE Probability** |
| --- | --- |
| **Any condition** | 4,7% |
| **Intraprostatic perineural invasion** | 28,9% |
| **Length capsular Involvement > 9.5 mm** | 35,4% |
| **Overall intraprostatic perineural invasion + length caps. Involvement > 9.5 mm** | 81,4% |

**ECE Probability = Exp(β) / [1 + Exp(β)]**, where **β = [-3.00 + 2.1*(IPNI) + 2.4*(LCI > 9.5 mm)]**

IPNI = intraprostatic perineural invasion; LCI = length of capsular involvement

**eTable 4 – Radiologists’ concordance**

| **Variables** | **ICC (IC 95%)** | **p-value** |
| --- | --- | --- |
| **Index Lesion size** | 0.842 (0.782 - 0.887) | **<0.001** |
| **PI-RADS v2** | 0.617 (0.495 - 0.715) | **<0.001** |
| **LIKERT** | 0.705 (0.604 - 0.784) | **<0.001** |
| **ESUR** | 0.644 (0.527 - 0.737) | **<0.001** |

| **Variables** | **ILS (mm)** | |
| --- | --- | --- |
|  | **Pearson’s correlation** | **p-value** |
| **ILS, L.G.** | 0.660 | **<0.001** |
| **ILS, J.H.** | 0.620 | **<0.001** |

ICC= intraclass correlation coefficient; ILS = index lesion size; mm= millimetres;

**eFigure 1 – ROC Curve analysis, comparison between final model and**

**PI-RADS/LIKERT/ESUR models**

**
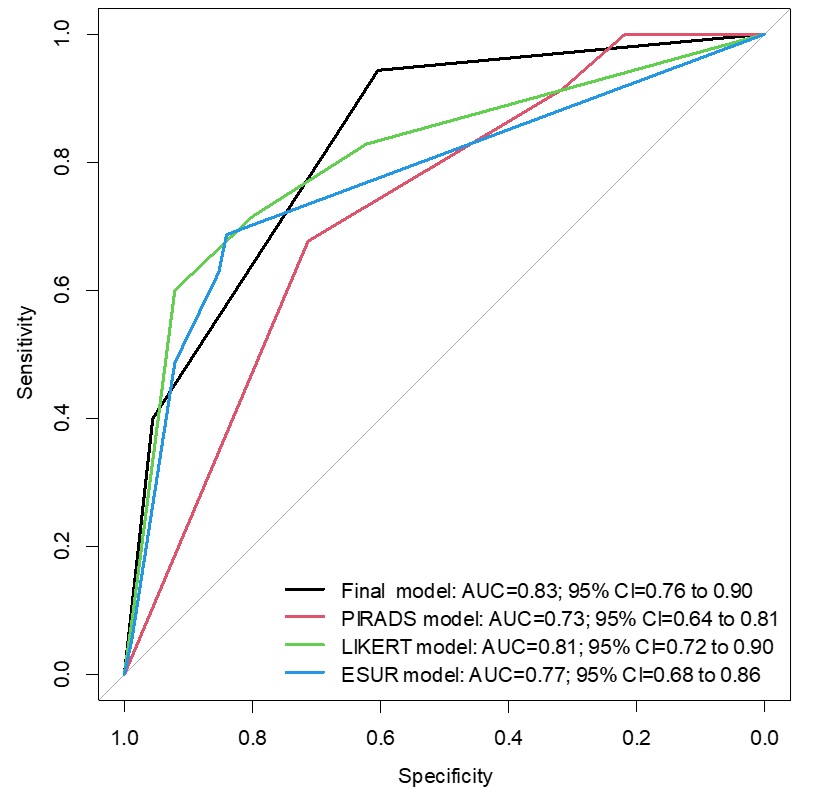
**
